# Expression profiling on soybean leaves reveals integration of ER- and osmotic-stress pathways

**DOI:** 10.1186/1471-2164-8-431

**Published:** 2007-11-23

**Authors:** André ST Irsigler, Maximiller DL Costa, Ping Zhang, Pedro AB Reis, Ralph E Dewey, Rebecca S Boston, Elizabeth PB Fontes

**Affiliations:** 1 Departamento de Bioquímica e Biologia Molecular, BIOAGRO, Universidade Federal de Viçosa, 36571.000 Viçosa, Minas Gerais, Brazil; 2 Molecular Core Facility, Department of Biology, Florida State University, Tallahassee, FL 32306-4370, USA; 3 Department of Crop Science, North Carolina State University, Raleigh, NC 27695, USA; 4 Department of Plant Biology, North Carolina State University, Raleigh, NC 27695, USA

## Abstract

**Background:**

Despite the potential of the endoplasmic reticulum (ER) stress response to accommodate adaptive pathways, its integration with other environmental-induced responses is poorly understood in plants. We have previously demonstrated that the ER-stress sensor binding protein (BiP) from soybean exhibits an unusual response to drought. The members of the soybean BiP gene family are differentially regulated by osmotic stress and soybean BiP confers tolerance to drought. While these results may reflect crosstalk between the osmotic and ER-stress signaling pathways, the lack of mutants, transcriptional response profiles to stresses and genome sequence information of this relevant crop has limited our attempts to identify integrated networks between osmotic and ER stress-induced adaptive responses. As a fundamental step towards this goal, we performed global expression profiling on soybean leaves exposed to polyethylene glycol treatment (osmotic stress) or to ER stress inducers.

**Results:**

The up-regulated stress-specific changes unmasked the major branches of the ER-stress response, which include enhancing protein folding and degradation in the ER, as well as specific osmotically regulated changes linked to cellular responses induced by dehydration. However, a small proportion (5.5%) of total up-regulated genes represented a shared response that seemed to integrate the two signaling pathways. These co-regulated genes were considered downstream targets based on similar induction kinetics and a synergistic response to the combination of osmotic- and ER-stress-inducing treatments. Genes in this integrated pathway with the strongest synergistic induction encoded proteins with diverse roles, such as plant-specific development and cell death (DCD) domain-containing proteins, an ubiquitin-associated (UBA) protein homolog and NAC domain-containing proteins. This integrated pathway diverged further from characterized specific branches of ER-stress as downstream targets were inversely regulated by osmotic stress.

**Conclusion:**

The present ER-stress- and osmotic-stress-induced transcriptional studies demonstrate a clear predominance of stimulus-specific positive changes over shared responses on soybean leaves. This scenario indicates that polyethylene glycol (PEG)-induced cellular dehydration and ER stress elicited very different up-regulated responses within a 10-h stress treatment regime. In addition to identifying ER-stress and osmotic-stress-specific responses in soybean (*Glycine max*), our global expression-profiling analyses provided a list of candidate regulatory components, which may integrate the osmotic-stress and ER-stress signaling pathways in plants.

## Background

Environmental stress conditions, such as water deficit, extremes of temperature and high-salinity, are major constraints for plant growth, crop productivity, and distribution. Different approaches to increase stress tolerance in plants have been undertaken, such as manipulating and reprogramming the expression of endogenous stress-related genes (for review see [1]). In general, strategies targeting expression of transcription factors and other regulatory genes have been effective by the consequent up-regulation of many downstream genes [2-5]. However, enhanced stress tolerance has also been achieved by changing the expression of a single downstream gene [6,7]. In this case, effective targets for engineering stress tolerance include genes involved in mechanisms that prevent intracellular stress build up, like the Na+/H+-antiporter gene [8], as well as those directly involved in cellular protection and repair, such as the antioxidant system and molecular chaperone genes [9-12]. The endoplasmic reticulum (ER) molecular chaperone Binding Protein (BiP), which provides cellular protection against ER stress in suspension cells and during seed germination, enhances tolerance to water dehydration when ectopically expressed in the model system tobacco [13]. Although the underlying mechanism for BiP-mediated increases in water-stress tolerance is not completely understood, the current knowledge of BiP function accommodates the argument that it may act in both mechanisms. In the first case, BiP would interact with downstream targets during water stress, in the second, it would activate transmembrane kinases that signal the ER stress [14-17].

As an ER-resident molecular chaperone, BiP has a major function to enable folding of newly synthesized secretory proteins by preventing misfolding or aggregation of folding intermediates [18-20]. In addition, BiP is involved in several other ER-associated cellular processes, such as protein co-translational translocation [21,22], modulation of calcium storage [23], ER-associated protein degradation [ERAD; [24,25]] and signaling ER stress by sensing alterations in the ER environment [14,26]. Any stress conditions that disrupt ER homeostasis and promote accumulation of unfolded proteins in the organelle trigger a cytoprotective signaling cascade that has been studied in detail in yeast and mammalian cells, and designated the unfolded protein response (UPR).

In yeast, ER stress is sensed by the luminal domain of the ER transmembrane protein kinase Ire1p, which, upon dimerization of the cytosolic domain and subsequent activation of its kinase and endonuclease domains, activates downstream events. The hallmark of this ER-stress response is the coordinated up-regulation of ER molecular chaperones leading to an increase in the ER protein processing capacity to prevent protein aggregation (for review see [20]). In mammals, the UPR is transduced by three distinct classes of ER transmembrane proteins: PERK, ATF6 and Ire1p homologues [27]. Upon activation, these proteins act in concert to trigger a transient attenuation of protein synthesis, degradation of misfolded proteins and up-regulation of ER folding functions.

The mammalian Ire1p homologues, designated Ireα and Ireβ, are structurally organized into a luminal-stress sensing domain, a transmembrane segment and cytosolic kinase/endonuclease domains [28]. The mammalian PERK is an eIF2-α kinase which inhibits protein translation in the early phase of the ER-stress response to maintain a proper balance between protein synthesis rate and ER processing capacity [29]. ATF6 is a transcription factor that under normal conditions is anchored to the ER membrane, with a C-terminal ER-stress sensing domain oriented to the ER lumen [30]. In response to ER stress, ATF6 is translocated to the Golgi, where it is specifically cleaved by S1P and S2P proteases to relieve its N-terminal transcription factor domain [31]. The cleaved ATF6 domain is targeted to the nucleus where it drives the coordinated up-regulation of a set of genes encoding ER chaperones and folding enzymes. The ER molecular chaperone BiP directly regulates the UPR by controlling the activation status of the three classes of transducers [14].

In plants, two Ire1p homologues have also been identified, but functional information is lacking and downstream components are yet to be identified [32]. Recently, an ER-stress induced leucine zipper (bZIP) transcription factor gene from Arabidopsis, designated *AtbZIP60*, has been shown to activate BiP and calnexin promoters through ER stress response element-like sequences [33]. AtbZIP60 is though to be anchored to the ER membrane under normal conditions but is released from the membrane upon sensing the ER stress by an unknown mechanism. The bZIP domain is translocated to the nucleus where activates the expression of molecular chaperones and its own expression. While the proposed initial trigger of AtbZIP60 activation by induced-conformational change resembles the mammalian ATF6 mechanism, its autoregulation is similar to that of XBP1 in mammalian cells.

Although little is known about the components of ER-stress signaling in plants, comprehensive genome-wide evaluations of the ER-stress-induced changes in gene expression have provided evidence that the major branches of the mammalian UPR are conserved in plants as well [34,35]. However, these transcriptional profiling analyses are restricted to the Arabidopsis model system and a global ER-stress response of crops remains to be determined. Likewise, genome-wide analyses and expression profiling studies in different plant species have revealed specific responses to wounding, drought, osmotic, cold and salt stresses, as well as the crosstalk between their signaling cascades, but these observations do not extend to include soybean responses [36-38]. Given the potential of BiP to regulate the UPR and the capacity of the BiP overexpressing plants to maintain leaf turgor under water deficit conditions [13,14], we reasoned that a genomic scale profile of the shared responses of ER and osmotic stresses would provide insights into the mechanism of BiP-mediated increases in osmotic balance under stress conditions. In addition to identifying ER-stress and osmotic-stress-specific responses in soybean (*Glycine max*), our global expression-profiling analyses provided a list of candidate regulatory components, which may integrate the osmotic-stress and ER-stress signaling pathways in plants.

## Results

**Figure 1:**
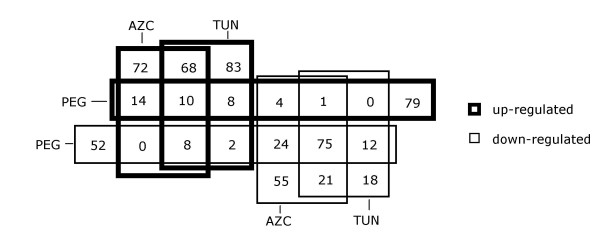
Venn diagram representing the distribution of specific and shared responses to TUN (10 μg/ml, 24 h), PEG (MW:8000 10%, 16 h) and AZC (50 mM, 16 h) treatments, as determined by microarray analysis. Determination of differential expression is described in Material and Methods. Thick boxes represent up-regulation and thin boxes represent down-regulation.

Using microarray slides containing amplified fragments of 5,760 soybean cDNAs, we conducted a broad survey to identify genes whose expression is affected by osmotic and ER stresses. In addition we expected to uncover any overlap in expression patterns that reflected integration of both stress-mediated signaling pathways. Three-week-old soybean plants were treated with either the ER-stress inducers L-azetidine-2-carboxylic acid (AZC) or tunicamycin, or the osmotic-stress inducer polyethylene glycol (PEG, which led to a loss of water of around 60–70% in replicate plants). Within the microarray, we included, as positive controls, ER-stress induced molecular chaperone genes, such as the soybean BiP isoforms, *A*, *C *and *D *[39,40]. Targets in the microarray slides were allowed to hybridize with pairs of Cy3- and Cy5-labeled cDNA probes from the following pairs of treated plants: tunicamycin and DMSO control, AZC and water control, PEG and water control. Two biological replicates and two technical replicates (dye-swap) were used for each treatment. The diagram shown in Figure 1 provides an overview of the microarray data showing the relative distribution of expression changes as shared and stress-specific responses.

### ER stress-specific response

A total of 306 genes were differentially expressed in response to tunicamycin treatment, with 179 being up-regulated and 127 down-regulated (Figure 1). In response to AZC treatment, 352 genes were differentially expressed, 172 up-regulated and 180 down-regulated. To specifically target the UPR-regulated genes, we considered the overlapping genes that significantly responded to both tunicamycin and AZC treatments. This overlapping transcriptional response consisted of 183 genes, 86 up-regulated and 97 down-regulated (Figure 1, see Additional files 1 and 2).

**Figure 2:**
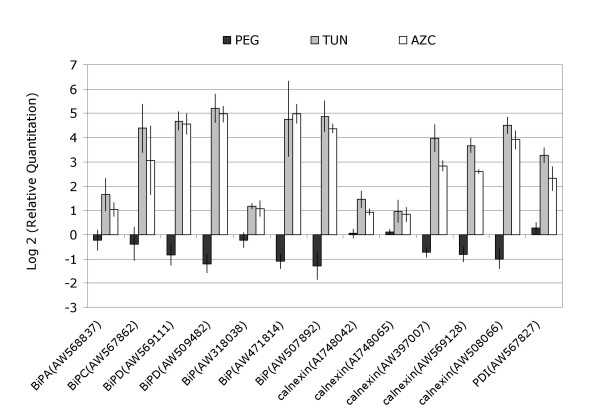
Effect of PEG, tunicamycin and AZC on the expression of *BiP*, *PDI *and calnexin genes (with accession numbers shown for each clone). The fold variation of gene expression (in relation to control treatment), as determined by microarray analysis, is presented in log_2 _scale (± SD, n = 4 biological and technical replicates).

Among the up-regulated genes, 17% represented genes of unknown functions. In the remaining set of genes for which some functional information was available, those that exhibited an ER-stress response signature predominated (see Additional file 2). More specifically, this up-regulated class of genes included those categorized as having a function in (i) protein folding (ii) ERAD and (iii) translational regulation. These results confirmed the activation of the ER-stress response pathway. In fact, the clones on the array with high homology to the known ER-resident molecular chaperones, BiP and calnexin, or the folding catalyst protein disulfide isomerase (PDI), were strongly up-regulated by both tunicamycin and AZC treatments (Figure 2).

**Figure 3:**
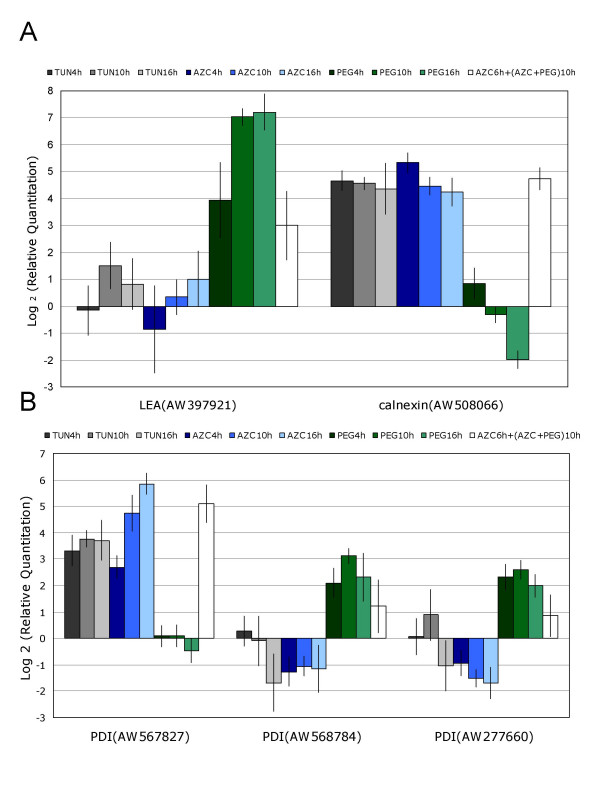
Time course of transcript induction by osmotic and ER stresses presented in log_2 _scale of gene expression, determined by real-time RT-PCR. Plants were treated with tunicamycin (gray), AZC (blue), PEG (green), or a combination of AZC and PEG (white) for the indicated period of time. A) Relative expression of representative genes of the response specific to ER (calnexin) or osmotic (SMP = LEA gene) stresses. B) Differential expression of the soybean PDI gene family members in response to tunicamycin and PEG treatments. GenBank Accession numbers are shown for each clone.

The expression of calnexin, an ER multi-functional protein involved in calcium homeostasis and protein folding, was used as a marker for ER-stress activation in a time-course experiment using real-time RT-PCR. Similar levels of induction of calnexin were observed between treatment with tunicamycin and AZC, possibly representing saturation in the expression (Figure 3A).

We found it particularly interesting that three cDNAs related to the PP2C and PP2A protein phosphatases were up-regulated by ER stress (see Additional file 1, AW471739, AW569267, AW509424), as would be expected if they represented genes involved in UPR signaling in stressed cells. Accordingly, the *PP2C*-related cDNA is a homolog of the yeast PP2C (AAB64644) that regulates the UPR by dephosphorylation of Ire1 [41], a transmembrane protein kinase/endoribonuclease that triggers the UPR [42,43].

The identification of wheat MLO (transmembrane domain mildew resistance) allelic variants as endogenous substrates of an ERAD-related quality-control machinery provided direct evidence that an ERAD-like mechanism operates constitutively in plants [44]. As part of the ER-stress response, the activation of this turnover mechanism has been observed in genome-wide analyses of *Arabidopsis *treated with inducers of ER stress and in a transcript-profiling assay of maize endosperm mutants that display a long-term ER stress response [34,35,45]. Here we also observed a tunicamycin and AZC up-regulated repertoire of putative ERAD-related genes in soybean, such as those encoding polyubiquitin, ubiquitin conjugating enzyme, alpha subunit of the proteasome, CDC48 and Derlin (see Additional file 1). These results further confirmed that, like in mammalian cells and in yeast, an ER stress-induced quality control mechanism in plants integrates the cellular response to conditions that alter protein folding in the ER.

During conditions of ER stress in mammals, a dynamic balance between the ER processing capacity and the protein synthesis rate is adaptively achieved through a transient and general down-regulation of protein translation, as a component of the ER-stress response [46]. The results of our microarrays were also effective in identifying a series of up-regulated genes related to ribosomal proteins (60S and 40S subunits; see Additional file 1) that might represent regulatory elements in protein translation. Likewise, we found that a translational inhibitor protein (AW508686), a eukaryotic translation initiation factor 3 subunit 10 (AW317679), and a translation elongation factor 1-gamma (AI960794), which are potentially regulators of protein translation, were also responsive to ER stress. Collectively, the global transcriptional analysis of soybean cDNAs in response to ER stressors clearly unmasked the major branches of the conserved ER-stress response, arguing favorably for a good sampling of the genomic representation on our array and for the biological validation of the global analyses.

### Osmotic stress-specific response

In response to osmotic stress caused by PEG treatment, a set of 116 up-regulated and 173 down-regulated genes was observed (Figure 1, see Additional files 3 and 4). Of particular interest were genes in two functional classes: genes directly involved in the stress response as cellular protectants, and regulatory genes involved in signaling events downstream of the osmotic stress response.

As representatives of the first class, we detected induction of late embryogenesis abundant (LEA) proteins, heat shock proteins (HSPs), senescence-related proteins, protease inhibitors, enzymes associated with osmolyte distribution, such as sugar transporters, and in osmoprotectant biosynthesis, such as Δ1-pyrroline-5-carboxylate synthase (P5CS), which is involved in proline biosynthesis [47,48], and sucrose synthase [49,50] (see Additional file 3). Also in this category, we observed up-regulation of anti-oxidative defense components, such as glutathione peroxidase and glutathione-S-transferase homologs, which are involved in detoxification and protection from reactive oxygen species [51,52] and lipid transfer proteins that could possibly function in the stress-damaged membrane repair system, or in the regulation of cellular membrane permeability by changing the lipid composition in response to stress [53,54]. As representatives of the second class, our microarray results detected the presence of two protein phosphatases, PP2C-like and PP1/PP2A PRL1-like coding cDNAs, and a serine/threonine-like protein kinase (see Additional file 3). We also detected PEG-induced members of the NAC family of transcription factors. NAC proteins induced by dehydration have been previously described in *Arabidopsis *and rice [5,55].

*LEA *genes were the most abundant among genes up-regulated by PEG treatment. We examined the osmotic stress response in more detail with real-time RT-PCR analysis of a gene encoding a putative LEA protein (Figure 3A). These data demonstrated that PEG treatment caused a gradual increase in induction of this *LEA *gene as a 15-fold increase in *LEA *mRNA level was detected at 4 h post-treatment, reaching 130- and 146-fold-changes at 10 h and 16 h, respectively. In contrast, the *LEA *gene did not exhibit a significant variation of expression in response to ER-stress inducers and thus could be used as an appropriate monitor of the level of osmotic stress in treated plants.

### The antagonistic response to ER stress and osmotic stress has an ER protein-folding signature

Different clones present in the soybean cDNA microarrays that likely encode ER-associated proteins, showed a differential and antagonistic regulation by ER and osmotic stresses. The most dramatic is within the PDI-like sequences where several family members were represented on the array. The protein disulfide isomerases (PDIs) belong to the superfamily of thioredoxin-domain-containing proteins that catalyze the formation of disulfide bonds and play an important role in protein folding. Within the TRX (thioredoxin) superfamily, they make a large gene family, designated *PDIL *(PDI-like proteins), encompassing disulfide isomerases and oxidoreductases that are associated predominantly with the protein secretory pathway in plants [56]. The biochemically and genetically characterized members of this family were first identified in plants as ER-resident proteins that were induced under ER-stress conditions [57]. Based on sequence comparison, we identified in our soybean arrays four thioredoxin domain-encoding cDNA fragments that were classified as members of the PDIL family. Two clones were highly and specifically induced by both tunicamycin and AZC treatments, whereas two others were only induced by PEG (see Additional files 1 and 3). Analysis of gene expression using real-time RT-PCR in a time-course experiment confirmed the differential regulation of the soybean PDIL gene family by osmotic and ER stresses and revealed that specific members of this family respond inversely to treatment with inducers of ER stress and PEG (Figure 3B). Sequence alignment and the expression pattern suggest to us that both PEG-induced clones refer to the same gene.

The finding that PEG treatment represses the tunicamycin-induced PDIL form extends to include other components of the ER protein processing machinery. An ATPase CDC48 homolog, *CDC48-like*, involved in ERAD was found to be up-regulated by ER-stress inducers, but repressed by PEG treatment (Table 1). Likewise, the *BiP *homolog clones as well as the PCR products of the soybean *BiP isoforms A, C *and *D *introduced in the arrays were inversely regulated by ER- and osmotic-stress (Figure 2). The cDNA homologs of the molecular chaperone calnexin were up-regulated by ER stress, but three calnexin cDNA fragments were significantly down-regulated by osmotic stress (Figure 2). We analyzed, by real-time RT-PCR, the kinetics of a calnexin homolog gene down-regulated by osmotic stress. The calnexin mRNA levels decreased gradually with the duration of treatment for 4, 10 and 16 h of exposure to PEG (Figure 3A). The general down-regulation of ER-molecular-chaperone genes by osmotic stress might reflect a general collapse and dysfunction of the ER under the severity of our PEG treatment rather than a specific biological phenomenon of gene regulation. To test this hypothesis we treated the soybean plants simultaneously with both PEG and AZC for 10 h and quantified the calnexin mRNA levels. Under PEG treatment, the ER stress agent AZC promoted calnexin induction to the same extent as did either AZC or tunicamycin treatment alone (Figure 3A). Likewise, for the ER-stress-induced *PDI *(Figure 3B), the simultaneous treatment of PEG and AZC promoted the same level of gene induction as did the AZC treatment alone. These results clearly demonstrated that the ER is functioning and capable of signaling and activating the UPR under the PEG treatment conditions. Thus, the down-regulation of ER folding activities in response to osmotic stress may be a specific cellular response of plant cells.

**Table 1:** Overlap of the ER stress and osmotic-stress transcriptional responses*

	Accession No. of Protein^a^	*e-value*^b^	Accession No. of Clone^c^	PEG^d^	*p-value*^e^	TUN^d^	*p-value*^e^	AZC^d^	*p-value*^e^
**Genes induced by all of the treatments**									
ATAF2 protein	BAC43493	8.E-31	AW459852	3.93	0.002	4.33	0.031	7.19	0.002
NAM protein	ABE79286	1.E-33	AW459732	2.81	0.008	3.31	0.019	5.27	0.001
N-rich protein	CAI44933	4.E-07	AI973541	2.76	0.005	3.39	0.009	4.33	0.012
N-rich protein	CAI44933	2.E-76	AW184865	1.93	0.008	2.14	0.012	2.42	0.003
ubiquitin-associated (UBA) protein	XP_466502	4.E-38	AW508375	3.05	0.000	3.12	0.024	2.59	0.014
eukaryotic translation initiation factor 5	P48724	2.E-65	AW472364	3.56	0.002	1.54	0.028	1.84	0.015
glutathione S-transferase	AAC18566	2.E-46	AW472161	3.83	0.003	3.19	0.002	2.75	0.002
glutathione S-transferase	AAG34800	1.E-48	AW397276	2.22	0.002	2.28	0.000	1.48	0.009
unknown			AW186110	2.58	0.005	3.17	0.003	9.56	0.000
unknown			AW508115	1.74	0.001	3.08	0.006	19.56	0.000
**Genes repressed by all the treatments**									
oxygen-evolving enhancer protein 1	P26320	3.E-39	AI941034	-3.27	0.000	-1.82	0.009	-3.07	0.001
thylakoid membrane phosphoprotein	NP_566086	1.E-21	AI960735	-1.93	0.004	-1.65	0.013	-2.03	0.011
NADPH-protochlorophyllide oxidoreductase	BAA21089	4.E-78	AW277941	-1.76	0.023	-2.08	0.009	-2.16	0.015
oxygen evolving enhancer protein 1 precursor	BAA96365	5.E-23	AW101019	-3.42	0.011	-2.24	0.001	-1.70	0.001
photosystem I chlorophyll a/b-binding protein	CAA45523	4.E-75	AW397435	-9.07	0.001	-4.96	0.009	-7.02	0.000
photosystem I chlorophyll a/b-binding protein	CAA45523	1.E-75	AW101657	-5.56	0.010	-2.38	0.015	-2.06	0.003
chlorophyll a/b-binding protein CP24 precursor	AAD27882	3.E-41	AI736217	-7.54	0.000	-5.72	0.003	-10.78	0.001
chlorophyll a/b binding protein type II	AAL29886	3.E-18	AI736285	-7.45	0.006	-3.69	0.048	-10.00	0.000
LHCII type III chlorophyll a/b binding protein	AAD27877	9.E-85	AW397809	-15.20	0.001	-10.48	0.004	-38.20	0.000
chlorophyll a-b binding protein	P13869	2.E-85	AW471940	-5.19	0.005	-4.23	0.016	-8.21	0.001
RuBisCO small subunit 1	CAA23736	7.E-53	AW278725	-4.74	0.003	-5.01	0.107	-58.93	0.000
photosystem II type I chlorophyll a/b-binding protein	AAA50172	1.E-64	AW472492	-28.79	0.002	-10.26	0.028	-31.02	0.000
oxygen-evolving enhancer protein 1	P26320	8.E-64	AW567782	-2.11	0.030	-1.65	0.050	-1.58	0.005
chlorophyll a/b-binding protein CP24 precursor	AAD27882	2.E-75	AW568341	-9.95	0.005	-6.83	0.004	-16.75	0.000
putative chlorophyll a/b-binding protein precursor	XP_482572	2.E-73	AW568620	-11.01	0.004	-6.02	0.004	-8.13	0.000
oxygen evolving enhancer protein 1 precursor	BAA96365	9.E-67	AW568090	-3.99	0.014	-4.53	0.037	-14.93	0.000
type II chlorophyll a/b binding protein	CAA57492	1.E-78	AW100823	-3.61	0.000	-2.93	0.024	-9.04	0.001
chlorophyll a/b-binding protein type I	AAQ54512	1.E-36	AW100631	-5.39	0.000	-2.70	0.006	-3.23	0.004
chlorophyll a/b-binding protein type III precursor	S04125	2.E-43	AI794678	-7.95	0.000	-3.36	0.037	-10.81	0.000
LHCII type III chlorophyll a/b binding protein	AAD27877	2.E-22	AW508739	-7.35	0.006	-3.23	0.007	-2.99	0.003
chlorophyll a/b-binding protein CP24 precursor	AAD27882	8.E-72	AW568252	-7.94	0.011	-3.40	0.002	-3.53	0.000
chlorophyll a/b-binding protein CP24 precursor	AAD27882	6.E-88	AW570380	-5.53	0.001	-5.23	0.012	-10.41	0.002
photosystem I subunit × precursor	AAL32043	4.E-46	AW277960	-4.65	0.014	-3.72	0.009	-4.81	0.000
oxygen-evolving enhancer protein 1	P14226	4.E-71	AW472001	-4.74	0.005	-3.13	0.028	-5.54	0.000
photosystem I psaH protein	AAQ21121	3.E-53	AW471851	-3.39	0.003	-2.98	0.002	-4.82	0.002
LHCII type III chlorophyll a/b binding protein	AAD27877	6.E-29	AW472547	-9.06	0.001	-3.54	0.019	-1.62	0.004
photosystem II reaction center W protein	CAA59409	1.E-28	AW471847	-2.55	0.015	-1.74	0.024	-1.49	0.002
photosystem II protein	AAM61462	6.E-07	AW508451	-4.91	0.009	-2.91	0.003	-1.72	0.004
photosystem I reaction center subunit III	AAD27880	1.E-82	AW508794	-5.25	0.002	-4.83	0.030	-33.31	0.000
geranylgeranyl hydrogenase	AAD28640	9.E-48	AW185978	-2.18	0.014	-1.89	0.003	-2.37	0.010
ultraviolet-B-repressible protein	AAS58469	1.E-29	AW317705	-5.24	0.003	-4.16	0.011	-10.16	0.000
glutamine synthetase precursor	AAK43833	4.E-66	AI736144	-2.28	0.010	-2.17	0.007	-2.19	0.010
myo-inositol-1-phosphate synthase	AAK72098	7.E-95	AI941146	-2.30	0.001	-1.71	0.011	-2.30	0.006
UDP-glucose 4-epimerase	Q43070	4.E-83	AI856802	-3.52	0.001	-2.64	0.001	-3.32	0.007
putative auxin-amidohydrolase precursor	CAG32961	1.E-18	AW278733	-4.23	0.001	-3.33	0.007	-8.72	0.000
granule-bound starch synthase Ib precursor	BAC76613	5.E-49	AW508018	-1.67	0.004	-2.21	0.031	-2.16	0.006
selenium binding protein	CAC67501	6.E-50	AW101647	-4.82	0.008	-4.94	0.021	-28.20	0.000
ATP-dependent helicase	NP_850847	1.E-58	AW570395	-3.97	0.003	-5.72	0.016	-12.70	0.000
microsomal omega-3 fatty acid desaturase	BAC87757	1.E-86	AI960953	-1.70	0.009	-1.71	0.012	-3.54	0.000
granule-bound starch synthase Ib precursor	BAC76613	4.E-65	AW472193	-7.20	0.008	-6.51	0.000	-17.58	0.000
carboxylic ester hydrolase	NP_177281	2.E-51	AW278929	-2.16	0.038	-3.73	0.023	-2.30	0.010
cinnamoyl-CoA reductase	AAY86360	5.E-60	AW508388	-3.11	0.007	-4.27	0.004	-5.60	0.000
transformer-SR ribonucleoprotein	CAA70700	3.E-38	AW568037	-3.07	0.011	-2.82	0.000	-7.39	0.000
putative cinnamoyl-CoA reductase	AAT39306	2.E-16	AW101559	-2.91	0.004	-3.01	0.005	-1.68	0.010
myo inositol 1-phosphate synthase	CAJ15162	5.E-56	AW100674	-4.42	0.000	-4.48	0.003	-5.52	0.001
aldose 1-epimerase-like protein	NP_566594	3.E-66	AW507799	-2.36	0.009	-2.74	0.012	-2.26	0.000
amino acid binding/ACT domain-containing protein	NP_565908	5.E-35	AW508692	-2.57	0.032	-1.75	0.002	-1.58	0.043
cytochrome P450 monooxygenase	AAD38930	8.E-55	AW507877	-1.86	0.009	-3.74	0.017	-2.98	0.027
palmitoyl-acyl carrier protein thioesterase	AAD01982	3.E-25	AW568268	-2.58	0.039	-1.71	0.021	-5.28	0.001
1-aminocyclopropane-1-carboxylate oxidase	AAX84675	3.E-86	AW508290	-6.79	0.010	-5.34	0.020	-11.62	0.000
plasma membrane polypeptide	CAB61742	1.E-40	AW459777	-1.83	0.028	-2.05	0.005	-1.67	0.016
acid phosphatase	CAA11075	8.E-20	AI930921	-3.69	0.000	-2.61	0.016	-4.07	0.001
ATP synthase gamma chain	CAA45150	5.E-80	AW186038	-4.15	0.002	-2.71	0.010	-5.77	0.000
putative leukotriene-A4 hydrolase	AAM91766	3.E-50	AW277270	-2.08	0.025	-1.67	0.019	-1.88	0.004
ATP synthase B' chain	CAA50520	4.E-24	AW471917	-2.94	0.008	-3.25	0.013	-7.91	0.001
granule-bound starch synthase Ib precursor	BAC76613	8.E-78	AW472190	-4.85	0.005	-5.28	0.001	-8.47	0.000
pepsin A	NP_196320	4.E-54	AW568189	-5.01	0.024	-2.34	0.003	-4.01	0.004
plastid ribosomal protein CS17	CAA77502	4.E-32	AW508645	-2.47	0.008	-2.16	0.005	-4.58	0.000
phosphoglycerate kinase	AAF85975	2.E-18	AW568791	-3.08	0.012	-2.44	0.009	-6.98	0.000
chitinase-like protein	BAC81645	1.E-38	AW508700	-2.20	0.015	-2.45	0.007	-2.81	0.001
unknown			AW508640	-1.80	0.017	-2.27	0.015	-1.61	0.033
unknown			AW570244	-2.03	0.004	-1.97	0.001	-2.81	0.001
unknown			AW598111	-2.16	0.005	-1.58	0.009	-2.58	0.013
unknown			AW508120	-4.73	0.001	-4.19	0.043	-19.62	0.000
unknown			AW100867	-2.40	0.007	-2.00	0.001	-1.65	0.016
unknown			AI941196	-1.85	0.003	-2.17	0.013	-2.01	0.000
unknown			AW164582	-1.64	0.006	-2.00	0.007	-3.58	0.001
unknown			AW471578	-2.03	0.025	-2.13	0.003	-3.51	0.000
unknown			AW508445	-3.28	0.008	-2.41	0.031	-1.67	0.020
unknown			AW507853	-3.02	0.014	-2.91	0.005	-6.71	0.000
unknown			AW569116	-3.17	0.002	-2.02	0.008	-1.47	0.038
unknown			AW471729	-2.02	0.037	-3.36	0.009	-2.07	0.008
unknown			AW568035	-10.74	0.006	-2.49	0.047	-1.79	0.001
unknown			AW101065	-3.07	0.008	-3.62	0.006	-3.41	0.033
unknown			AW568660	-6.56	0.002	-2.69	0.002	-3.33	0.000
**Genes induced by TUN and AZC but repressed by PEG**									
CDC48-like protein	AAP53974	5.E-71	AW509037	-1.70	0.009	10.57	0.000	5.78	0.001
calnexin homolog precursor	BAD81043	9.E-78	AW569128	-1.76	0.044	12.82	0.000	6.09	0.000
calnexin homolog precursor	Q39817	9.E-59	AW397007	-1.67	0.005	15.69	0.001	7.16	0.000
calnexin homolog precursor	Q39817	1.E-82	AW508066	-2.00	0.015	22.76	0.000	15.03	0.000
BiP isoform D			AW569111	-1.78	0.029	25.60	0.000	23.72	0.000
BiP isoform D	AAK21920	3.E-101	AW509482	-2.30	0.008	37.13	0.000	31.41	0.000
BiP	BAD95470	1.E-76	AW471814	-2.16	0.023	27.22	0.034	31.90	0.000
BiP	BAD95470	5.E-68	AW507892	-2.43	0.022	29.42	0.001	20.75	0.000
**Genes repressed by TUN and AZC but induced by PEG**									
unknown			AW186103	1.74	0.003	-1.77	0.049	-1.72	0.005

* Additional files 1, 2, 3, 4, 5, 6 contain Microarray results that were used to generate Table 1. ^a ^Protein annotations obtained from BlastX using cDNA clone sequence against GenBank. ^b ^Expected values obtained by BlastX. ^c ^Clone accession number in the GenBank. ^d ^Fold variation in gene expression converted from means of the log_2_ratio (treated/control channel) from plants treated with PEG, TUN or AZC and their respective controls. ^e ^probability values obtained from the *t-test*.

### Modest overlap of the ER-stress and osmotic-stress transcriptional responses

An overlap of the osmotic-stress and the ER-stress responses is represented by 10 up-regulated genes (see Additional file 5), 75 down-regulated genes (see Additional file 6), 8 ER stress-induced but osmotically repressed genes, and 1 osmotically stress-induced but ER stress-repressed gene (Figure 1, Table 1). Thus, only about 10% of the genes up-regulated by either ER or osmotic stress in our survey population were induced by both treatments. In contrast, a substantial overlap in the genes down-regulated by ER and osmotic stresses was observed, with about 50% and 75% of the genes being in the sets affected by osmotic stress and ER stress, respectively. These results represent a much larger down-regulation of transcripts by ER stress than those reported previously ([34,35]; see Additional files 2 and 6). Likely, these results reflect substantial differences in experimental design and conditions, including cDNA library origin, plant background, stringency of stress conditions, plant species, setting up and processing of microarrays.

While 25% of the repressed genes that were ER-stress specific were predicted to be related to the secretory pathway (see Additional file 2), the remaining 75% were co-repressed genes that seem to represent a general response of plants to abiotic stresses, as they reflect an inhibition of photosynthesis and development. In fact, a common effect of osmotic and ER stresses revealed by the microarray analysis was a general decrease in the expression of photosynthesis-related genes, including genes that encode the oxygen-evolving enhancer protein, chlorophyll a/b binding protein, small subunit of RuBisCO, NADPH-protochlorophyllide oxidoreductase (involved in chlorophyll biosysnthesis), a thylakoid membrane phosphoprotein and others. There are at least 32 redundant clones involved in photosynthesis that are down-regulated by all three treatments (Table 1). Consistent with photosynthesic inhibition was our observation of three starch synthase homolog cDNAs, and two clones related to cinnamoyl-CoA reductase, a lignin synthesis-related gene being repressed by both stresses (Table 1). Another class represented in the co-repressed category included members involved in hormone biosynthetic pathways, such as 1-aminocyclopropane-1-carboxylate oxidase (*ACC*, AW508290), involved in ethylene biosynthesis [58], an IAA-amino acid conjugate hydrolase (AW278733), which regulates the level of the auxin indole-3-acetic acid [IAA; [59]], geranylgeranyl hydrogenase and cytochrome P450 monooxygenase that participate in the gibberellin (GAs) biosynthetic pathway [60,61].

**Figure 4:**
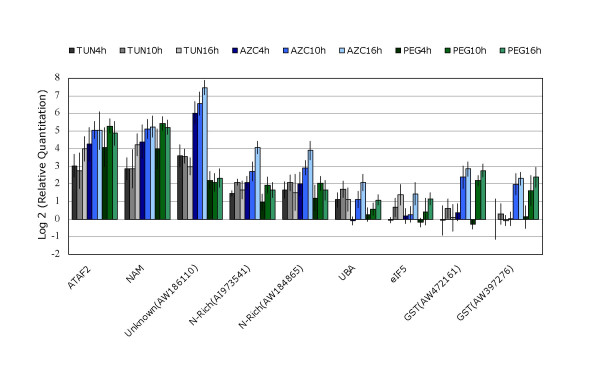
Time course of transcript induction of the co-up-regulated genes by tunicamycin, AZC and PEG treatments. The fold variation (± SD, n = 3 biological replicates) showed in log_2 _scale of gene expression was determined by real-time RT-PCR, from plants treated with tunicamycin (gray), AZC (blue), or PEG (green), for the indicated period of time. GenBank Accession numbers of certain clones are presented to help clone identification.

The overlap in genes that shared the up-regulated response revealed two cDNA fragments that encode putative transcription factors belonging to the NAM family (NAC and ATAF2 homologs), two clones that encode DCD-domain-containing proteins (N-rich proteins), two encoding glutathione-S-transferase, a *UBA *protein gene, an eukaryotic translation initiation factor 5 (*eIF5*) gene and two cDNAs whose function is unknown (Table 1). A more precise analysis using real-time RT-PCR confirmed a significant induction of co-up-regulated transcripts by tunicamycin, AZC or PEG treatment, except for glutathione-S-transferase (GST) mRNAs which did not show induction by tunicamycin (Figure 4).

If these genes had a role in regulating this branch of the pathway, they would be predicted to be induced early. We tested this possibility with a time-course experiment. Real-time RT-PCR assays during induction demonstrated that the *NAC*-containing proteins *ATAF2 *and *NAM *exhibited an early kinetics of induction, consistent with their putative role as transcriptional factors (Figure 4). Four-hour treatments were sufficient to saturate their expression, which remained high for the duration of the experiment. A similar kinetic pattern was observed for the *N-rich *DCD-domain transcripts AI973541 and AW184865, which were strongly induced at 4 h and reached maximum accumulation at 10 h post treatment. Accordingly, the *N-rich *(AW184865) gene has been shown to be rapidly induced during the hypersensitive response in soybean [62]. In contrast to the early induction of the *NAC *and *N-rich *related genes by ER and osmotic stresses, the induction of the remaining co-up-regulated genes occurred with delayed kinetics (Figure 4). The induction of the *UBA*, *eIF5*, *GST *(AW472161) and *GST *(AW397276) transcripts was initially detected by 10 h post-treatment and continued to increase through the 16 h time point. The kinetic pattern of the co-up-regulated genes clearly defined a class of early response genes that may have regulatory functions and delayed genes that may exhibit protective functions.

**Figure 5:**
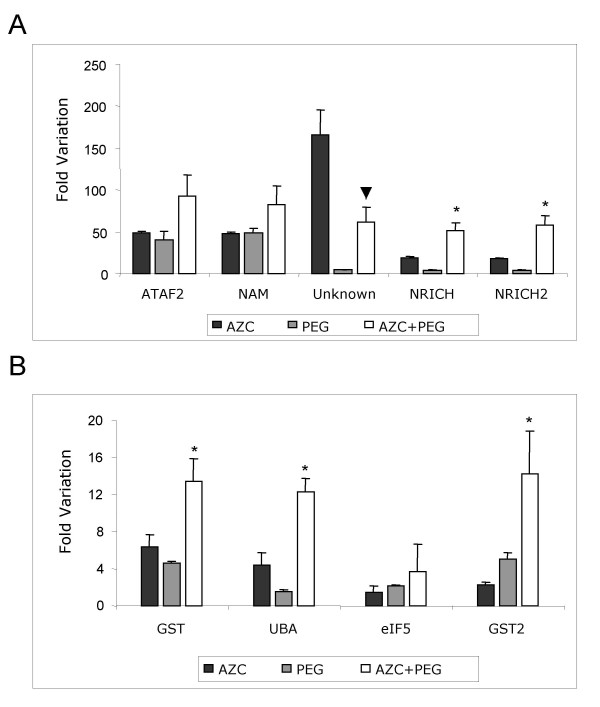
Synergistic induction of gene expression by the combination of PEG and AZC treatments. Values from fold variation of gene expression are the mean ± SD from three biological replicates, as determined by real-time RT-PCR. Plants were treated for 16 hours with AZC, for 10 hours with PEG, or for 6 hours with AZC only, and then 10 hours with a combination of AZC and PEG. Values for AZC 6 h + (AZC+PEG) 10 h are relative to H_2_O control treatment for 16 hours (± SD, n = 3 biological replicates). Non-additive responses are indicated by asterisks (synergism) and arrowhead (epistasis).

To examine directly the interactions of ER and osmotic stress on the co-up-regulated response, we analyzed whether the combination of AZC and PEG treatments promoted an additive increase in expression (Figures 5A and 5B). Five of the nine co-induced genes that we examined (asterisks) were induced by both stimuli in a more than additive fashion. Thus, the ER-stress and osmotic-stress signaling responses are integrated in a synergistic, convergent manner at the gene activation level. In this non-additive response, we also observed integration of the pathways by epistasis as PEG treatment reduced drastically the effect of AZC on the induction of the unknown gene (Figure 5A).

## Discussion

Using a cDNA microarray potentially representing approximately 5,700 soybean gene tags, our expression profiling in response to ER stress and osmotic stress provided an unprecedented view of the overlapping transcriptional responses to ER stress and osmotic signaling. However, in searching for crosstalk between these two signaling pathways, we detected a much larger change for the UPR-regulated transcripts than was reported in *Arabidopsis *[34,35]. Given that a plant is constantly adapting to its environment and its physiological status will impact the overall response to stress [36], one may consider that some of the changes reported here are associated with specific conditions of our experimental design. For instance, to induce ER stress both AZC and tunicamycin were directly taken up through the cut petiole of young soybean plants and vascularly translocated to their leaves. Excision of the petiole would not be expected to elicit a wound response [63] and any effects due to tissue treatments would be accounted for by inclusion of untreated cut petiole controls to prevent wounding-specific changes from being included as variation in ER-stress responses. To extend the candidate gene list of the overlapping ER-stress and osmotic signal responses, we used a relative low stringency cutoff criterion for variation in expression (close to a 2-fold change in expression level). Additionally, we used an experimental design based on two-biological plus two-technical replicates to minimize detection of random and technical variations. The validity of the approach was supported by the fact that our results revealed the major branches of the ER-stress response as well as the major osmotically regulated changes reported in other surveys [34-38]. In addition, the coordinate induction of a subset of genes by both ER and osmotic stresses was confirmed by real time RT-PCR (Figure 4).

Our results indicated that genes encoding ER chaperones and folding catalysts, such as BiP, calnexin and PDI, were antagonistically affected by the PEG-induced osmotic signal and activation of the UPR. While the UPR-mediated up-regulation of the ER molecular chaperones is a conserved feature in eukaryotic cells (for review see [20]), coordinate down-regulation of these proteins by PEG-induced dehydration has not been previously described. In fact, previous studies describing drought- or osmotic-stress responses have focused on just a small subset of ER molecular chaperones. For instance, in spinach, drought stress has been shown to reduce the BiP mRNA level, whereas in soybean and tobacco, a subset of BiP transcripts has been shown to be up-regulated by PEG-induced osmotic stress, water deficit or ABA treatment [13,42,64,65]. The apparent contradiction of these results has been explained as a function of the plant background in which the BiP basal level and the cellular secretory activity would signal the necessity of BiP up- or down-regulation under drought. More recently, genomic scale information on stress-induced changes has allowed a more in-depth view of the scenario for reprogramming plant gene expression as the result of interaction of the plant with the environment. A recent wide-genomic analysis of PEG-specific changes in maize clearly demonstrated that a large fraction of down-regulated transcripts are represented by protein biosynthesis-related genes [66]. These results are not surprising as PEG-induced cellular dehydration is expected to slow down protein synthesis. Under these conditions, a repression of ER folding activities by the osmotic signal would permit the ER protein processing capacity to be balanced with the low rate of protein synthesis. Our results showing a coordinate down-regulation of ER molecular chaperones in response to PEG treatment fit quite well with this current model of coupling ER protein processing capacity to the rate of protein synthesis [67]. However, whether the decrease in the ER protein processing capacity is a primary response to the osmotic signal or a consequence of the limitation in the overall protein synthesis rate under cellular dehydration remains to be determined.

The present ER-stress- and osmotic-stress-induced transcriptional studies demonstrate a clear predominance of stimulus-specific positive changes over the shared response (5.5% of the total up-regulated genes). This scenario indicates that PEG-induced cellular dehydration and ER stress elicited very different up-regulated responses within a 10-h stress treatment regime. In contrast, we observed a much larger overlap of the down-regulated response. From the 195 clones significantly down-regulated in the microarray analysis, 75 cDNAs (38%) were found to be down-regulated by all the stress treatments. These possibly represent a general stress response. In fact, a large fraction of the genes down-regulated in all treatments consist of photosynthesis-related genes, such as chlorophyll a/b binding protein, components of photosystems I and II and the small subunit of RUBISCO, as well as genes associated with development, for example, those encoding enzymes involved in hormone biosynthetic pathways. Recently, a cDNA microarray analysis revealed that photosynthesis-related genes were down-regulated by PEG treatment of maize seedlings [66]. Likewise, similar studies in other plant species have demonstrated that inhibition of photosynthesis is a common general response to drought, cold, high salinity and ABA [37,68].

Our data provide evidence that the up-regulated response common to all treatments was indeed an integrated pathway reflecting crosstalk between the UPR and osmotic stress signaling. A combination of the ER stress- and osmotic stress-induced treatments promoted a synergistic effect on the induction level of the common up-regulated genes although to a different extent for various genes (Figure 5, asterisks). These results indicate that information transfer between the signaling pathways occurs through the shared, integrated response with the potential to alter or to intensify the output of the different pathways. Furthermore, they suggest that the ER stress and osmotic signaling pathways are likely to converge on the co-regulated target genes at the level of gene activation. Based on these observations, we considered as components of the integrated pathway only the subset of the co-regulated genes that were synergistically induced by the simultaneous treatment of the soybean plants with AZC and PEG (Figures 5A and 5B, asterisks). Comparison of the overlapping positive responses at different time points classified the integrative genes as having early or delayed effects. The early genes include the homolog cDNAs for *ATAF2 *and *NAM*, which belong to the *NAC *gene family of trans-acting factors (for review see [69]). Several members of this family of plant specific DNA-binding proteins have been shown to exhibit transcriptional activation [5,55,70-72]. The delayed genes consisted of functional genes which may exhibit cytoprotective properties, such as UBA (ubiquitin-associated) domain protein, possibly involved in the ubiquitin pathway. Osmotic and ER stresses are known to generate reactive oxygen species that trigger the induction of the antioxidant system [73-75]. These results provide a critical framework for future studies on the elucidation of the pathways integrating ER stress and osmotic signals.

With respect to the underlying mechanism of BiP-mediated increases in water deficit tolerance that provided the foundation for pursuing these studies, the results of the microarray analysis highlighted relevant insights. The observed PEG-mediated down-regulation of ER molecular chaperones may imply that, unlike tunicamycin, PEG treatment does not cause protein misfolding in the ER. This finding argues against the need to maximize the ER protein processing capacity for cellular recovery from the osmotic stress. Therefore, under the PEG-induced stress conditions of our experiments, which mimic drought stress in soybean, an ectopic increase of ER molecular chaperone activities per se could not counteract the global deleterious effects of the osmotic stress. In view of these observations, it is reasonable to assume that the protective role of BiP against water dehydration may not be associated with its molecular chaperone activity, but rather it may be linked to its regulatory role as a sensor of the ER stress signal [14,26]. Like in mammalian cells, the induction of BiP in plants has been shown to block ER stress signals [76]. How might a block in the ER stress signal by high BiP concentrations affect osmotic signaling? The finding that these signaling pathways converge upon the integrative genes to potentiate the cellular response provides the molecular link that would permit the flow of the integrated information to be controlled by a regulator of either one of the stress signals. Additional experiments will be required to elucidate the physiological consequences of activation of the integrated pathway and to determine how or if manipulation of BiP levels might affect the response.

The integrative genes, such as *ATAF2 *homolog and the *N-rich *genes, have been linked to the pathogen response and programmed cell death [PCD; [77,78]]. Overexpression of the Arabidopsis *ATAF2 *gene in transgenic lines led to repression of a number of pathogenesis-related protein genes, whereas their levels were increased in *ATAF2 *knock-out lines [77]. ATAF2 belongs to the *NAC *gene family that is represented by 109 members in the Arabidopsis genome [79] and 20 of these are present in the leaf senescence dbEST [80]. Additionally, many groups have reported expression of *NAC *genes in senescing leaves [80-84] and a NAC transcription factor (NAM-B1) isolated from wheat has been shown to regulate leaf senescence [85]. As for the *N-rich *genes, they encode a DCD (development and cell death) domain which is thought to be involved in the hypersensitive response and programmed cell death [62,78]. Based on the putative roles of the integrative genes, the possibility that the integrated pathway might transduce a PCD signal generated by prolonged ER stress and osmotic stress warrants further investigation.

## Conclusion

The present ER-stress- and osmotic-stress-induced transcriptional studies demonstrate a clear predominance of stimulus-specific positive changes over shared responses on soybean leaves. This scenario indicates that PEG-induced cellular dehydration and ER stress elicited very different up-regulated responses within a 10-h stress treatment regime. In contrast, we observed a much larger overlap of the down-regulated response with a predominance of phothosynthesis-related and developmental genes that may represent a general response to stress. In addition to identifying ER-stress and osmotic-stress-specific responses in soybean (*Glycine max*), our global expression-profiling analyses provided a list of candidate regulatory components, which may integrate the osmotic-stress and ER-stress signaling pathways in plants. A combination of the ER stress- and osmotic stress-induced treatments promoted a synergistic effect on the induction level of the common up-regulated genes, indicating that the ER stress response integrates the osmotic signal to potentiate transcription of shared target genes. These studies thus provide the groundwork for further investigations into the physiological relevance of activation of the integrative pathway and into the involvement of the ER-stress sensor BiP in the response.

## Methods

### Plant growth and stress treatments

For the microarray experiments, soybean (*Glycine max*) seeds (cultivar Dare) were germinated in soil (MetroMix-360, Scotts, Marysville, OH) in a growth chamber with a day/night cycle of 9/15 h at 26°C/22°C. The aerial portions of three-week-old plants were excised below the cotyledons and directly placed into 15 ml of 10% (w/v) polyethylene glycol (PEG; MW 8000, Sigma, St. Louis, MO), 10 μg/ml tunicamycin (Sigma) or 50 mM L-azetidine-2-carboxylic acid (AZC, Sigma) solutions. The first trifoliate leaves were harvested after 16 h of PEG or AZC treatment (water control) and after 24 h of tunicamycin treatment (DMSO control, Sigma), then immediately frozen in liquid N_2 _and stored at -80°C until use. In all experiments two independent biological replicates were used.

For the real-time RT-PCR experiments, soybean seeds (cultivar Conquista) were germinated in soil and grown in greenhouse conditions (avg. 21°C, max. 31°C, min. 15°C) under natural conditions of light, relative humidity 70%, and approximately equal day and night length. The first trifoliate leaves of three-week-old plants were excised and fed, via the petiole, solutions that induce the osmotic (10% PEG w/v) or ER stress responses (10 μg/ml tunicamycin or 50 mM AZC). After treatments for the times indicated in figure legends, the stressed trifoliate leaves and their untreated counterparts were immediately frozen in liquid N_2 _and stored at -80°C until use. To avoid PEG interference in AZC uptake during the combined treatments we pre-treated the plants with AZC for six hours, when PEG was added for an additional 10 hours. Treatment with PEG and AZC simultaneously for 16 hours was found to give similar results. Each stress treatment and RNA extraction were replicated in three independent experiments.

### Generation of soybean microarrays

The microarray slides consisted of 5,760 amplified cDNA fragments from soybean libraries prepared from RNA of developing seeds [86]. ESTs from these libraries were placed into contigs to identify unigenes [86], therefore a low redundancy in our set of clones is expected. The cloned cDNA fragments were amplified with M13 primers, purified using PCR Cleanup Filter Plates (Millipore, Bedford, MA), and eluted in water (according to the manufacturer's protocol). An aliquot of each amplified fragment reaction was separated through a 1% (w/v) agarose gel and visualized with ethidium bromide to assess size, quality and quantity. The purified PCR products were transferred to 384-well plates, and diluted with an equal volume of DMSO (Sigma). Finally, the PCR products were arrayed onto UltraGAPS slides (Corning, Corning, NY) using a 417 TM Arrayer (Affymetrix, Santa Clara, CA), cross-linked by exposure to UV light at 250 mJ and baked at 75°C for 2 h.

Isoforms of the ER stress-related molecular chaperone BiP were amplified with gene-specific primers (see Additional file 7) and included in the arrays. They consisted of three soybean isoforms A, C and D [39,40].

### RNA extraction and labeled cDNA preparation

Total RNA was extracted from frozen leaves with TRIzol (Invitrogen, Carlsbad, CA) according to the instructions from the manufacturer, and further purified through silica columns. The quality and integrity of the RNA was monitored by spectrophotometry and agarose gel electrophoresis, respectively. For the microarray hybridizations, 10 μg of total RNA were reverse-transcribed with the SuperScriptTM Indirect cDNA Labeling System (Invitrogen) in the presence of cyanine-3-dUTP (Cy3-dUTP) or cyanine-5-dUTP (Cy5-dUTP; Amersham Biosciences, Piscataway, NJ) according to the manufacturer's instructions. RNA samples from each biological replicate were labeled twice, once with each dye, to control for dye-specific effects on the hybridizations.

For the real-time RT-PCR, 2 μg of total RNA were treated with DNase (Promega, Madison, WI) and fractionated through RNA purification columns (Qiagen, Valencia, CA). Reverse transcription was carried out using M-MLV reverse transcriptase (Invitrogen) and oligo-dT (18, IDT, Coralville, IA) primers (according to the protocol of the manufacturer). Prior to the real-time RT-PCR assays, the quality of the cDNA was assessed by PCR with gene-specific primers for ubiquitin associated protein (UBA; AW508375) to test for genomic DNA contamination, as these primers amplify a larger fragment size from genomic DNA than from cDNA.

### Microarray hybridization, scanning and data analysis

Soybean cDNA microrrays were subjected to a similar hybridization protocol as described by [87]. Briefly, the microarray slides were incubated for 45 min in 50 ml of a pre-hybridization buffer 5× SSC, 0.1% (w/v) SDS and 1% (w/v) of BSA (all from Sigma), washed sequentially in ultra-pure water and iso(2)-Propanol (Fisher, Waltham, MA), and air-dried. Slides were then incubated with Cy3- and Cy5-labeled cDNA (20 μl) from treated and control samples for 20 h at 42°C in a water bath protected from light. The hybridization buffer consisted of 0.5% SDS (w/v), 5× SSC, 5× Denhardt's, 50% (v/v) formamide, 0.5 μg/μl denatured calf thymus DNA (all from Sigma) and 0.5 μg/μl polyA RNA (Amersham Biosciences). Following incubation, slides were washed sequentially in three steps in the following solutions: (1) 1× SSC, 0.2% (w/v) SDS, (2) 0.1× SSC, 0.2% (w/v) SDS, and (3) 0.1× SSC.

Microarray slides were scanned at the Cy3 (530 nm) and Cy5 (650 nm) wavelengths with a ScanArray 4000 laser fluorescent scanner (Packard Bioscience, Perkin Elmer, Wellesley, MA), at a laser power of 100% and photomultiplier tube (PMT) gain of 75%. The image analysis and calculation of mean background-subtracted intensity of the spots were performed using QuantArray software version 2.2 (PerkinElmer). Normalization based on the LOWESS algorithm [88] and data analysis were performed using Genespring software version 7.2 (Agilent Technologies, Santa Clara, CA).

Genes were considered differentially expressed if they met both of two criteria. The first was an average fold change greater than 1.63 for PEG, 1.50 for TUN and 1.46 for AZC. These values were calculated based on the mean of gene expression ratio for each treatment plus two standard deviations (expression ratio values above 2 were not included for this calculation). This filtering criterion based on cut-off values of fold-change provided a pool of candidate genes differentially expressed. The second criterion, based on the t-test, was used to determine the statistical significance of the differences observed for the selected genes. The null hypothesis of the t-test [(mean of log2ratio)/SE] was rejected at 5% of probability. Additionally, genes with greater differences in expression (above 2 fold) in both biological replicates (but not necessarily in both technical replicates) were considered differentially expressed. A low stringency in our microarray data analysis was applied because relative expression of genes co-regulated by stress treatments was more accurately measured by real-time RT-PCR.

Annotations and Arabidopsis homologs of the soybean clones were assigned based on the top BlastX predictions against the GenBank [89] and The Arabidopsis Genome Initiative databases [90]. For most of the corresponding proteins there is not a demonstrated biochemical function; therefore, we refer to them as "like" proteins.

*Note: *The data discussed in this publication have been deposited in NCBIs Gene Expression Omnibus [91] and are accessible through GEO Series accession number GSE8992.

### Real-time RT-PCR data analysis

For the quantitative RT-PCR assays, sequences of cDNA and primers are listed in Additional file 7. Analysis of expression of calnexin, an ER-stress responsive gene, and seed maturation protein PM30, a drought-induced gene, were used as positive controls for the respective stress treatments.

To select an endogenous control gene for data normalization in real-time RT-PCR analysis, we analyzed three genes encoding histone H2A, 60S ribosomal protein L30, and RNA helicase, which had been chosen because they had low and consistent expression ratios in the microarray results. The RNA helicase was used to normalize all values in the real-time RT-PCR assays, because it exhibited the lowest variation in expression values among treatments.

Real-time RT-PCR reactions were performed on an ABI7500 instrument (Applied Biosystems, Foster City, CA), using SYBR^® ^Green PCR Master Mix (Applied Biosystems). The amplification reactions were performed as follows: 2 min at 50°C, 10 min at 95°C, and 40 cycles of 94°C for 15 sec and 60°C for 1 min. To confirm quality and primer specificity, we verified the size of amplification products after electrophoresis through a 1.5% agarose gel, and analyzed the Tm (melting temperature) of amplification products in a dissociation curve, performed by the ABI7500 instrument.

Fold variation in gene expression was quantified using the comparative Ct method: 2^-(ΔCtTreatment – ΔCtControl), which is based on the comparison of expression of the target gene (normalized to the endogenous control) between experimental and control samples.

## Authors' contributions

ASTI carried out the experiments, the statistical analysis of the data and drafted the manuscript. MDLC and PAB assisted directly the qRT-PCR assay. PZ assisted the generation of soybean microarrays. RED designed the microarray experiments and critically reviewed the manuscript. RSB designed the experiments and critically reviewed the manuscript. EPBF designed the experiments and drafted the manuscript. All authors have read and approved the manuscript.

## Supplementary Material

Additional file 1 Table S1. Genes up-regulated by TUN and AZC treatments, but not by PEG treament. The table lists and functionally categorizes genes up-regulated specifically by TUN and AZC treatments with the corresponding fold variations (in red). Click here for file

Additional file 2 Table S2. Genes down-regulated by TUN and AZC treatments, but not by PEG treatment. The table lists and functionally categorizes genes down-regulated specifically by TUN and AZC treatments with the corresponding fold variations (in blue). Click here for file

Additional file 3 Table S3. Genes up-regulated by PEG treatment, but not by AZC and TUN treatments. The table lists and functionally categorizes genes up-regulated specifically by PEG treatment with the corresponding fold variation (in red). Click here for file

Additional file 4 Table S5. Genes down-regulated by PEG treatment, but not by AZC and TUN treatments. The table lists and functionally categorizes genes down-regulated specifically by PEG treatment with the corresponding fold variation (in blue). Click here for file

Additional file 5 Table S6. Genes up-regulated by TUN, AZC and PEG treatments. The table lists and functionally categorizes genes up-regulated by all three treatments (TUN, AZC and PEG) with the corresponding fold variations (in red). Click here for file

Additional file 6 Table S6. Genes down-regulated by TUN, AZC and PEG treatments. The table lists and functionally categorizes genes down-regulated by all three treatments (TUN, AZC and PEG) with the corresponding fold variation (in blue). Click here for file

Additional file 7 Table S7: Genes analyzed by qRT-PCR and primers for soybean BiP isoforms. The table lists the genes analyzed by qRT-PCR and their corresponding primers. Click here for file
